# Robotic radiosurgery system patient‐specific QA for extracranial treatments using the planar ion chamber array and the cylindrical diode array

**DOI:** 10.1120/jacmp.v16i4.5486

**Published:** 2015-07-08

**Authors:** Mu‐Han Lin, Iavor Veltchev, Sion Koren, Charlie Ma, Jinsgeng Li

**Affiliations:** ^1^ Department of Radiation Oncology University of Maryland School of Medicine Baltimore MD USA; ^2^ Department of Radiation Oncology Fox Chase Cancer Center Philadelphia PA USA; ^3^ Department of Radiation Oncology Davidoff Cancer Center, Rabin Medical Center‐Beilinson Campus Petah Tikva Israel

**Keywords:** pretreatment verification, CyberKnife, SBRT

## Abstract

Robotic radiosurgery system has been increasingly employed for extracranial treatments. This work is aimed to study the feasibility of a cylindrical diode array and a planar ion chamber array for patient‐specific QA with this robotic radiosurgery system and compare their performance. Fiducial markers were implanted in both systems to enable image‐based setup. An in‐house program was developed to postprocess the movie file of the measurements and apply the beam‐by‐beam angular corrections for both systems. The impact of noncoplanar delivery was then assessed by evaluating the angles created by the incident beams with respect to the two detector arrangements and cross‐comparing the planned dose distribution to the measured ones with/without the angular corrections. The sensitivity of detecting the translational (1–3 mm) and the rotational (1°–3°) delivery errors were also evaluated for both systems. Six extracranial patient plans (PTV 7–137 cm^3)^ were measured with these two systems and compared with the calculated doses. The plan dose distributions were calculated with ray‐tracing and the Monte Carlo (MC) method, respectively. With 0.8 by 0.8 mm^2^ diodes, the output factors measured with the cylindrical diode array agree better with the commissioning data. The maximum angular correction for a given beam is 8.2% for the planar ion chamber array and 2.4% for the cylindrical diode array. The two systems demonstrate a comparable sensitivity of detecting the translational targeting errors, while the cylindrical diode array is more sensitive to the rotational targeting error. The MC method is necessary for dose calculations in the cylindrical diode array phantom because the ray‐tracing algorithm fails to handle the high‐Z diodes and the acrylic phantom. For all the patient plans, the cylindrical diode array/ planar ion chamber array demonstrate 100%/>;92%(3%/3 mm) passing rates. The feasibility of using both systems for robotic radiosurgery system patient‐specific QA has been demonstrated. For gamma evaluation, 2%/2 mm criteria for cylindrical diode array and 3%/3 mm criteria for planar ion chamber array are suggested. The customized angular correction is necessary as proven by the improved passing rate, especially with the planar ion chamber array system.

PACS number: 29.40.‐n

## I. INTRODUCTION

The robotic radiosurgery system (CyberKnife, Accuray Incorporated, Sunnyvale, CA), which utilizes numerous noncoplanar beams to achieve a superior plan dose conformality, resembles both multiple isocenter stereotactic radiosurgery and intensity‐modulated radiotherapy. This technique has been increasingly employed for the stereotactic body radiotherapy (SBRT) to treat the extracranial lesions, such as spine, prostate, lung, and liver. The recent development of a multileaf collimator (MLC)‐based CyberKnife further emphasizes its application on extracranial treatment.

A typical CyberKnife treatment consists of 100–300 beams arranged in wide spatial angles to offer a sharp dose fall‐off in the target region. CyberKnife plan delivery is fully image‐guided, which involves the real time orthogonal X‐ray image acquisition, analysis, and signal feedback to the treatment manipulator arm and the robotic couch. Based on our experience, most SBRT extracranial treatments utilize the IRIS cone, which is a variable‐aperture collimator using two sets (upper and lower banks) of six tungsten segments to create 12‐sided variable field sizes. This IRIS field size changes the size during the treatment. All these factors, together with the much higher dose per fraction than those in conventional fractionation, have contributed to the complexity of CyberKnife dose delivery and pose a more proportionate need for patient‐specific quality assurance (QA) prior to patient treatment

Though “patient‐specific end‐to‐end testing” has been recommended in ACR‐ASTRO Practice Parameter for the Performance of Stereotactic Radiosurgery,^(1)^ there is no consensus about the pretreatment patient‐specific QA for CyberKnife treatments. Guidelines of the periodic QA procedures for the CyberKnife system have been described in the AAPM Task Group (TG) reports number 135^(2)^ and 142.^(3)^ However, a formal guidance for the CyberKnife patient‐specific QA is unestablished as of the time of this work. A comprehensive patient‐specific QA should cover plan calculation, plan transfer, as well as the delivery. In current CyberKnife clinical practice, the independent MU calculation (i.e., hand calculation) is commonly utilized for the CyberKnife pretreatment QA. But this approach does not verify the delivery. To validate the plan transfer and the delivery, pinpoint ion chamber measurements are employed by some CyberKnife treatment centers. This appears to be a reasonable approach to validate the intracranial treatments since the treatment targets are mostly small and the point dose is representative to the target dose. However, for the extracranial SBRT treatment, single point‐dose validation may not be sufficient, since the planning target volume (PTV) is relatively large and the passing/failing of a single dose point does not represent the passing/failing of the entire dose volume. Two‐dimensional (2D) measurements are more informative and representative of the entire dose distribution than the point‐dose measurements.

Due to the superior spatial resolution, films have been widely used for 2D relative dosimetry of the external radiation treatment beam, especially for treatment machine commissioning and periodical QA. It was also investigated by different groups for patient‐specific QA for treatment with IMRT^4^, ^5^ and CyberKnife,^6^, ^7^ especially when the radiochromic film became available for external beam therapy (EBT). However, it is still not widely used in clinic for routine patient‐specific QA because of the handing and the sensitivity, accuracy, and reproducibility dependency on the proper choice and calibration of the densitometry system. Currently, the electronic dosimetry system is still more popular in clinic use for patient‐specific QA because of its easy handling and the reproducibility. The electronic dosimetry systems with various geometry configurations and detector types introduced for routine patient‐specific QA for linac‐based treatments include planar ion chamber/diode arrays^8^, ^9^, ^10^, ^11^, ^12^, ^13^, ^14^, ^15^ (e.g., MatriXX, IBA Dosimetry; Octavius 729, PTW; MapCHECK, SunNclear Corp.), cylindrical diode arrays^16^, ^17^, ^18^, ^19^, ^20^, ^21^, ^22^ (ArcCHECK, Sun Nuclear Corp), and two diode arrays embedded in a cross‐plane fashion in a cylindrical phantom^(23)^ (Delta^4^, ScandiDos AB, Uppsala, Sweden). In contrast to the measurements for the linac delivery, which are mostly in a coplanar manner with larger field sizes, the measurements for the CyberKnife delivery can be more challenging since the delivery involves numerous narrow unflattened photon beams at a wide range of spatial angles. The beams can travel in between detectors and cause loss of measurement data. The responses of the dosimetry systems with respect to the delivery errors are, therefore, expected to be different.

The 3%/3 mm gamma passing rate metric is commonly adopted for intensity‐modulated radiotherapy (IMRT) and volumetric‐modulated arc therapy (VMAT) patient‐specific QA.^24^, ^25^ With considering the technical differences between the linac and the CyberKnife deliveries, it is desirable to investigate whether this 3%/3 mm metric is adequate for CyberKnife patient‐specific QA, and whether different detector systems require different gamma criteria.

This study aims to investigate and compare the feasibility of using the cylindrical diode array and the planar ion chamber array for CyberKnife patient‐specific QA. We focus on extracranial cases, since the current design of the planar or the cylindrical detector geometry is not practical for measuring the intracranial cases, which involves a lot of vertex beams. Alternating the phantom setup orientation would allow measuring the vertex beams, but it will require at least two verification plans corresponding to different phantom setup orientations and the tricky setup poses a concern of triggering the machine clearance safety interlock.

We investigate the feasibility of phantom setup with the kV orthogonal imaging and the noncoplanar beam measurements for the two detector arrays. The sensitivity of detecting various setup and delivery errors with respect to the planar/cylindrical geometry and the ion chamber/diode detectors is also evaluated. Finally, the criteria for passing/failing the patient‐specific QA are discussed, based on the verification results of selected clinical CyberKnife plans (target sizes 7 to 137 cm^3)^.

## II. MATERIALS AND METHODS

The MatriXX planar ion chamber array (Iba Dosimetry America, Inc., Bartlett, TN) and the ArcCHECK cylindrical diode array (Sun Nuclear; Melbourne, FL) were employed in this study. The MatriXX consists of 1020 ion chambers arranged in a crisscross manner with a 7.62 mm distance between individual chambers.^(26)^ The ion chamber size is 4.5 mm (diameter) ×5 mm (height). The ArcCHECK consists of 1386 N‐type diodes arranged in a helical arrangement with a 10 mm distance between individual diodes inside a 26.59 cm cylindrical phantom. The diode size is 0.8 mm (width) ×0.8 mm (length) ×0.03 mm (height).^(27)^

Table 1 summarizes the recruited cases, which covered various PTV sizes (7 cm^3^ to 137 cm^3)^ and five of the most common extracranial treatment sites: prostate, pelvis, spine, liver, and lung. The CyberKnife plans were generated by the MultiPlan (Accuray Inc.) treatment planning system^(28)^ with the cone sizes ranged from 7.5 mm to 40 mm.

The QA plans were generated by registering the original treatment plan on to the detector geometry. There are two dose calculation algorithms available in the MultiPlan: the ray‐tracing method and the Monte Carlo (MC) method. In this study, the QA plans were calculated with both ray‐tracing and MC methods,^28^, ^29^ respectively, for each case and detector combination. The two planned doses were compared with the measured dose, respectively. However, the data in the results section were all based on the MC dose calculations, which gave more correct dose estimations for both detector geometries.^(30)^ High resolution (0.9×0.9×1.25 mm) was used for final dose calculation and the statistical uncertainty for MC calculation is 1%.

**Table 1:** The selected cases

*Case*	*Site*	*PTV Size (cm^3^)*	*Cone Size (mm)*	*Number of Beams*
1	Prostate	137	20, 30, 40	258
2	Pelvis	106	15, 35, 60	162
3	Liver	69	10,30,40	218
4	Spine	46	15, 30	177
5	Lung 1	23	10, 15, 25	205
6	Lung 2	7	7.5, 10, 12.5	98

### A. CyberKnife measurements with detector arrays

**Figure 1:**
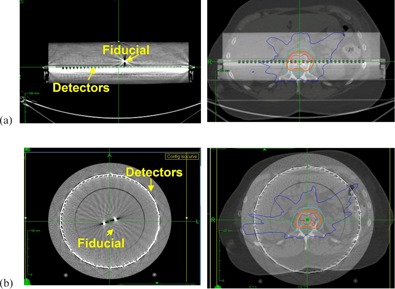
Fudicial marker setup and the CyberKnife plan registration for (a) the MatriXX and (b) the ArcCHECK systems.

CyberKnife plan deliveries require imaging setup with a pair of orthogonal kilovoltage (kV) X‐ray images and a given tracking method based on either bony anatomy, fiducial makers, or soft tissue contrast (e.g., lung tumor). The fiducial marker tracking appears to be the only suitable choice for the phantom measurements. Therefore, we attached four gold seed fiducial markers in a noncoplanar manner for both systems (Fig. 1).

The fiducial markers should be placed closely to the ‘targeted’ region to ensure correct setup. Therefore, four fiducial markers were placed between the phantom slab and the detector plate for the MatriXX system (Fig. 1(a)) and another four markers were attached in the central plug of the ArcCHECK system (Fig. 1(b)). The treatment plans were registered to the center of the phantom when verification plans were created.

The absolute dose calibrations were performed based on the ion chamber absolute dose measurements of the 60 mm cone for both systems. For the ArcCHECK measurement, the default real‐time corrections for the field size and beam angle have to be switched off since these corrections were configured based on the linac setup. The angular correction was not applied to the initial MatriXX measurements. For the absolute dose calibration and the output factor measurement, the center of an ion chamber/diode detector was aligned to the center of the beam for both systems to ensure the correct measurements of small cone sizes.

### B. Output factor

To evaluate the ability of measuring the small and unflattened beams for both systems, the dose output for all IRIS field sizes were measured by both the MatriXX and the ArcCHECK systems, respectively. The output factor is the ratio of the dose reading of the center detector for an individual cone to the dose reading of the 60 mm cone.^31^, ^32^ The measured output factors were compared with the commissioning data measured with a pinpoint ion chamber (PTW, Freiburg, Germany).

### C. Impact of noncoplanar beam delivery

The CyberKnife delivery sequence is defined in the .XML file exported from the MultiPlan system, which is similar to the RTP file in the linac TPS. This .XML contains the source point and the target point defining the beam direction, the monitor unit (MU), and cone size of individual beams.

An in‐house program was developed to apply beam‐by‐beam angular corrections to the measurements. This program reads the XML file. The spatial angle between the beam and the detector surface was extracted based on the planar/cylindrical geometry of the MatriXX/ArcCHECK systems. Subsequently, the movie files of the MatriXX/ArcCHECK measurements were imported and segmented into each beam delivery. The angular correction factors were applied to dose of each by postprocessing the initial measured data. The angular correction factors for MatriXX system were obtained from measurements with a linac 6 MV beam from different directions.^(33)^ The angular correction factors for ArcCHECK system were provided by the manufacturer.^(18)^ The correction factors for both systems were determined by the measurements of a reference detector (e.g., ion chamber) and an ion chamber/diode at two different locations: a given incident angle and 0° incident angle. The angular correction was then determined by the ratio of doses at those two positions.

(1) C=IiRiI0R0

As shown in Eq. (1), *C* is the angular correction factor, and $I_{0}$ and $I_{i}$ represent dose readings of the ion chamber/diode detector of the detector array under 0° and a given (i) incident beam angle, representatively; and $R_{0}$ and $R_{i}$ represent dose readings of the reference detector under 0° and a given (i) incident beam angle, representatively.

The incident angles of individual beams were extracted from each CyberKnife plan to assess the impact of the noncoplanar beam delivery with respect to the planar and cylindrical detector geometry. Gamma evaluation (3%/3 mm) was performed to quantify the agreements between the planned dose and the measured dose with and without angular corrections, respectively. The evaluation was performed for all the pixels with dose higher than 10% of their own maximum dose at the detector level. The gamma passing rates for measurements with and without angular corrections were compared against each other to demonstrate the dosimetric consequence of the noncoplanar beam delivery. Angular corrections are applied for both systems for the following tests.

### D. Sensitivity of error detection

We categorized the delivery uncertainties of the CyberKnife delivery into: 1) systematic error, which refers to the setup uncertainties and the daily output drift, and these errors would present in the entire plan delivery; 2) random error, which refers to the treatment manipulator (gantry) positioning errors and the output fluctuations that randomly occurred during the delivery.

#### D.1 Systematic error

The setup accuracy of CyberKnife delivery relies on the orthogonal kilovoltage (kV) X‐ray setup images and the automatic 6D couch movements. The imperfect calibration and maintenance of the kV X‐ray imaging system and the robotic couch system can lead to a systematic setup error in the treatment delivery. In order to evaluate the sensitivity of error detection for both systems, we manually introduce various levels of systematic uncertainties to the dosimetry systems after the initial setup performed by the orthogonal images. The systematic translational setup errors were mimicked by shifting the couch in superior‐to‐inferior directions (SI, i.e., axial), anterior‐to‐inferior (AP, i.e., sagittal), and the right‐to‐left (RL, i.e., coronal) for 1 to 3 mm. The systematic rotational error is mimicked by introducing 1° to 3° rotation to the couch. The minimum shift of 1 mm and rotation of 1° were selected since they are experimentally achievable and they are the tolerance set by AAPM Task Group Report 142^(3)^ for SRS/SBRT machine QA. For the purpose of testing two dosimetry systems, which are not originally designed for CyberKnife measurements, the maximum shift was set as 3 mm.

In addition, the daily output drift was mimicked, changing the MU of the treatment plan by 1%, 2%, and 3%, respectively. The dose distributions measured by both systems were compared to the planned ones. Gamma passing rates for both 2%/2 mm and 3%/3 mm criteria with respect to the introduced errors were plotted and analyzed for both systems.

#### D.2 Random error

We assume the random errors of the beam direction and the output during treatment can be described as a Gaussian distribution. We randomly sample the error from the Gaussian distribution and then intentionally introduced them in the plan by changing the targeting point and the output of the beam in the ‘fine‐tune’ mode of the planning system (MultiPlan).

For a given beam, the magnitude of error was randomly sampled from the Gaussian distribution and introduced to a randomly selected dimension (x, y, z) of the targeting point position. Similarly, a random output variation sampled from a Gaussian distribution was added to the MU of each individual beam. We studied three scenarios by changing the width (sigma) of the Gaussian distributions. For the targeting error, the sigma was set as 0.5 mm, 1 mm, and 2 mm. For the output fluctuation, the sigma was set to 3%, 5%, and 10%. The means of the Gaussian distributions were 0 and the sampling ranges were ± 3 sigma.

The dose distributions measured by both systems were compared to the planned ones. Gamma passing rates for 2%/2 mm and 3%/3 mm criteria with respect to the introduced errors were plotted and analyzed for both systems.

### E. CyberKnife patient plan validation

The ability of the two systems for CyberKnife patient‐specific QA was evaluated with six clinical body‐site SBRT cases treated with CyberKnife. QA plans were generated on both the MatriXX and the ArcCHECK phantoms and delivered for measurements. The measured dose distributions were evaluated with the gamma criteria of 3%/3 mm and 2%/2 mm, respectively, to further investigate the proper evaluation criteria for patient specific QA with both systems.

## III. RESULTS

### A. CyberKnife measurement setup with KV images

**Figure 2:**
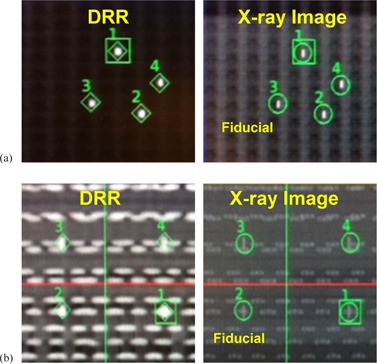
The DRR and the on‐board kilovoltage X‐ray images for (a) the MatriXX and (b) the ArcCHECK systems.

Figure 2 shows the DRRs and the kV X‐ray images taken during the treatment setup. The fiducial markers can clearly be seen in the MatriXX system (Fig. 2(a)), which mostly is composed of lower Z materials. In contrast, the high‐Z diodes of the ArcCHECK system created noticeable artifacts on the kV images and make the image‐guided setup more challenging. Thus, our homemade fiducial marker plug was designed to have the four fiducial markers being perpendicular to the diodes so that the fiducial markers can be distinguished on the setup images (Fig. 2(b)).

### B. Output factor

**Figure 3:**
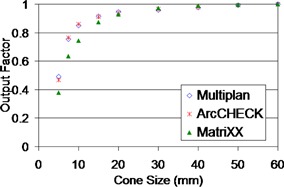
The output factors measured by a pinpoint ion chamber (i.e., commissioning data in MultiPlan) for the ArcCHECK and the MatriXX systems.

The output factors measured by both systems agreed with the commissioning data of the MultiPlan system for the larger cone sizes (Fig. 3). With the small active detector size $\left(0.8\times 0.8\;{\text{mm}}^{2}\right)$ of the diodes, a difference of 0.023 (0.468 ArcCHECK vs. 0.491 MultiPlan for 5 mm cone) is observed between ArcCHECK and commissioning data. While the 4.5 mm wide ion chamber size caused spatial averaging in the MatriXX measurements, a difference of 0.055 (0.858 vs. 0.913 for 15 mm cone) to 0.113 (0.378 vs. 0.491 for 5 mm cone) output factor difference is found between the pinpoint chamber measurement and the MatriXX measurements.

### C. Impact of noncoplanar beam delivery

**Figure 4:**
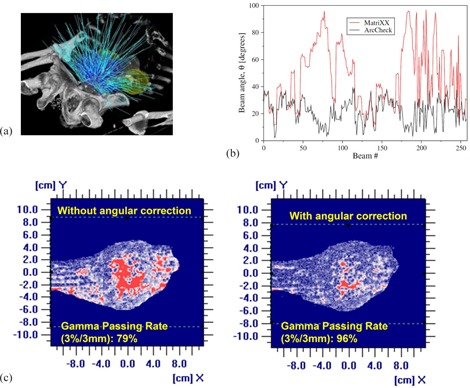
The beam arrangement of the prostate plan (case 1) (a); (b) the incident beam angles of each beam for the MatriXX and the ArcCHECK systems; (c) the gamma value map of the 2D dose comparisons between the TPS and the MatriXX measurements without and with the angular correction.

All the cases demonstrate a similar trend for the noncoplanar delivery test. A prostate case (case 1) containing the largest number of beams orientations is shown here as an example. Figure 4(b) shows the incident angle of each beam to the MatriXX (red) and to the ArcCHECK (black) detectors unfolded from the CyberKnife plan. The CyberKnife delivery creates various incident beam angles (10° to 90°) to the planar detector geometry (MatriXX). However, for the cylindrical detector arrangement (ArcCHECK), the incident beams are more perpendicular to the detectors (<40°). The maximum angular correction for a given beam is 8.2% for the MatriXX and 2.4% for the ArcCHECK system, respectively.

When the angular correction was applied to each beam with the in‐house program, significant improvement on the gamma test passing rate is observed for the MatriXX measurements. As demonstrated in Fig. 4(c), the MatriXX measurement for this prostate plan exhibits a 79.0% gamma passing rate (3%/3 mm) without the angular corrections and the red color indicates the failing points. The passing rate increases to 96.1% after the angular corrections is applied. However, the gamma passing rates without the angular corrections for the ArcCHECK drops from 100% and 98.8% only, which indicated that the angular effect is less significant for ArcCHECK.

### D. Sensitivity of error detection

#### D.1 Systematic error

**Figure 5:**
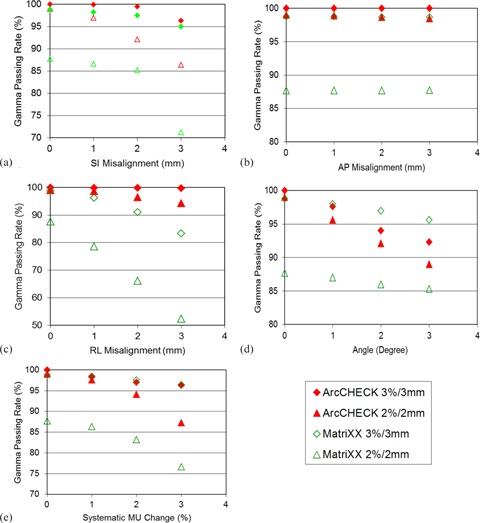
The gamma passing rates under various systematic delivery uncertainties: (a) SI misalignments, (b) AP misalignments, (c) RL misalignments, (d) rotational misalignments, and (e) systematic output change for the MatriXX and the ArcCHECK systems.

Figure 5 shows the gamma passing rates under 3%/3 mm and 2%/2 mm criteria with respect to various systematic errors applied on both systems. Both systems exhibit comparable response to the SI misalignments (Fig. 5(a)). However, with the smaller detector size, ArcCHECK seem to be more sensitive to small shifts (<2 mm) as demonstrated by the 6.9% gamma passing rate drop (from 99.0% at 0 mm to 92.1% at 2 mm), while the MatriXX system does not demonstrate a significant gamma passing rate variation when the SI misalignment is less than/equal to 2 mm. Neither of these systems is sensitive to the AP misalignments (Fig. 5(b)), since most of the beams are from AP and AP oblique directions (Fig. 4(a)) and AP misalignment mainly creates SSD variations rather than location change for the CyberKnife beams from anterior direction. For the RL misalignments, the MatriXX demonstrates a higher sensitivity of detecting this error (Fig. 5(c)) because the dose distribution location changes with the RL misalignments. For the rotational misalignments, a significant gamma passing rate reduction is observed for the ArcCHECK measurements (Fig. 5(d)). For systematic MU or output change, both systems have comparable error detecting sensitivity (Fig. 5(e)).

#### D.2 Random error

**Figure 6:**
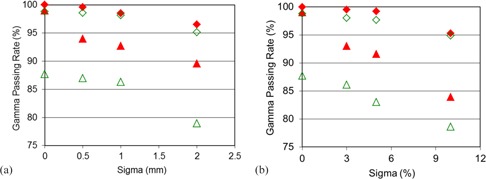
The gamma passing rates under various random delivery uncertainties: (a) random manipulator targeting errors and (b) random MU fluctuations, for both the MatriXX and the ArcCHECK systems.

Figure 6 shows the gamma passing rates under 3%/3 mm and 2%/2 mm criteria with respect to various random errors applied on both systems. As seen in Fig. 6(a), the two systems demonstrate a comparable sensitivity of detecting the targeting error, while the ArcCHECK exhibits a better response to small targeting error. ArcCHECK is also more sensitive to the output fluctuation randomly occurred for different beam angles.

### E. CyberKnife patient plan validation

Table 2 summarizes the gamma passing rates under 3%/3 mm and 2%/2 mm criteria for all cases. The angular corrections are applied for both systems. The ArcCHECK shows 100% passing rate under 3%/3 mm criteria and over 96% passing rate under 2%/2 mm criteria. Overall, the passing rates for all cases are lower for the MatriXX measurements. The passing rates drop below 90% when the 2%/2 mm criterion is applied.

**Figure 7:**
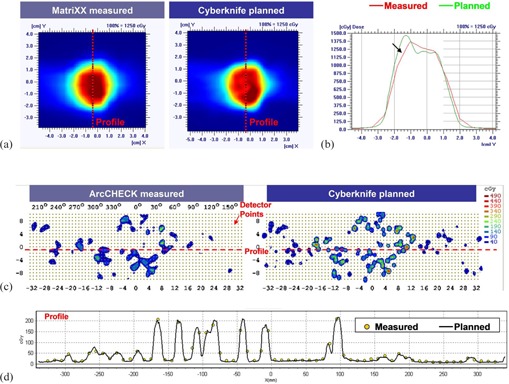
The 2D dose comparisons and the profile dose comparisons between the TPS (MultiPlan) and the MatriXX ((a), (b)) and the ArcCHECK ((c), (d)) measurements.

Figure 7 shows the dose comparison results of the two systems for the Lung 2 case, which has the smallest PTV size (7 cm^3)^. Figure 7(a) shows the measured and calculated 2D dose distributions for the MatriXX system. Figure 7(b) shows the dose profiles along the central axis of the detector plane. As seen in the profile comparison, the 4.5 mm wide ion chamber detectors of the MatriXX system causes a noticeable spatial averaging effect on the measured dose.

Figures 7(c) and (d) show the comparison of the ArcCHECK measured and TPS calculated dose distributions. Though some beams could be partially or entirely missed because of the 10 mm detector spacing, the small active detector size $\left(0.8\times 0.8\;{\text{mm}}^{2}\right)$ of the diodes ensures a correct dose measurement whenever the diode is irradiated by the beam, as demonstrated in the profile comparison (Fig. 7(d)).

**Table 2:** The gamma passing rates under 3%/3 mm and 2%/2 mm criteria

		*ArcCHECK*		*MatriXX*	
*Case*	*Site*	3%/3 mm	2%/2 mm	3%/3 mm	2%/2 mm
1	Prostate	100	99.0	96.1	79.6
2	Pelvis	100	96.3	97.7	86.3
3	Liver	100	97.4	91.3	75.8
4	Spine	100	99.2	94.4	78.3
5	Lung 1	100	96.8	94.4	80.5
6	Lung 2	100	99.6	94.0	71.8

## IV. DISCUSSION

This work investigates the feasibility of using two dosimetric systems with different detector types and detector arrangements for the CyberKnife SBRT dose measurements. To our knowledge, this is the first work addressing the patient‐specific QA for a nonisocentric, noncoplanar beam delivery modality with 2D dosimetry system. The current work focused on the cone‐based SBRT deliveries. However, the results of this work could also be applied to the MLC‐based CyberKnife treatment.

**Figure 8:**
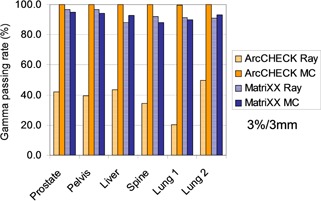
The gamma passing rates of the comparisons between the ArcCHECK/MatriXX measurements and the ray‐tracing/MC dose calculations.

Two dose calculation algorithms are available in the MultiPlan: ray‐tracing and Monte Carlo. We've performed the dose calculations with both algorithms for both systems. It is worth to mention that the ray‐tracing algorithm fails to handle the high‐Z diodes and the acrylic phantom material of the ArcCHECK, and results in an unfavorable Gamma passing rate (20% to 43%) for the CyberKnife plan measurements, as seen in Fig. 8. Therefore, MC calculation is necessary for the ArcCHECK. In contrast, with the water‐equivalent detectors and phantom, the MatriXX system does not exhibit as large effect.

It is well known that the reading of the diode, the nontissue‐equivalent detectors, has significant dependency on the beam incident angles.^(34)^ Due to the chamber shape and design, wiring, and additional buildup/backscatter materials, the MatriXX system also demonstrates angular dependency to the incident beam angles.^(35)^ The results of this work have shown that the noncoplanar beam delivery results in an even more significant dosimetric impact to the MatriXX system than the ArcCHECK. This owes to the different detector arrangement of the two systems. Comparing to the cylindrical detector arrangement in the ArcCHECK which allows the incident beams to remain almost perpendicular to the diodes, creating a smaller angle on the diodes, the planar detector arrangement in the MatriXX results in larger beam incident angles on the ion chambers (Fig. 4(b)). Hence, a significant improvement is observed in the MatriXX when the angular corrections are applied (Fig. 4(c)). It can be conclude that angular corrections are essential for the MatriXX measurements.

Feygelman et al.^(36)^ compared the “X” (Delta^4)^ and “O” (ArcCHECK) detector geometries and concluded that different detector systems truly measures different things. In this work, we further investigated the ability of catching setup and delivery errors for cylindrical and planar detector geometry (Fig. 5). It has been shown that both systems exhibit comparable sensitivity of detecting SI and AP misalignments, though the ArcCHECK is more sensitive to small misalignments due to its smaller detector size. However, MatriXX appears to be more sensitive to the RL misalignment, while ArcCHECK is more sensitive to rotational error. This is because the RL misalignments introduce a shift to the entire detector plane, as well as the measured 2D dose distribution for the planar detector geometry. But for the cylindrical geometry, only the detectors in the anterior and posterior of the system are shifted by equal distance in the measured 2D dose distribution when the RL misalignment is applied. In addition, with detectors at both the beam entry side and the exit side and the smaller detector size, ArcCHECK is more feasible to detect the slight dose variation introduced by the rotational misalignment. It is obvious that, if the MatriXX is placed in the axial orientation, it will be more sensitive to the AP misalignment. But one has to keep safety concerns in mind.

CyberKnife dose delivery stability degrades with the decreased MU per beam and cone size.^(37)^ A primary purpose of patient‐specific QA is to evaluate whether the plan can be correctly delivered. Systems capable of picking up the output error are preferable. It is observed that MatriXX and ArcCHECK have similar response to the systematic output drift. But the ArcCHECK system is more sensitive to the output fluctuation that randomly happened during the delivery (Fig. 6(b)). This is because the peripheral distributed detectors measure the dose mostly from an individual beam and the output fluctuation of different beams would be delivered to different detection areas. The output fluctuation of the individual beam is, therefore, easy to be detected. However, the planar distributed detectors of MatriXX measuring the accumulated dose in target region, the output fluctuations of individual beams and their effects would be canceled out in the measured dose distribution.

There is no consensus about the gamma passing rate for CyberKnife patient‐specific QA. Gallo et al.^(7)^ utilized EBT film for CyberKnife SBRT plan delivery measurement and reported a 93.3% to 98.9% and 96.1% to 99.5% gamma passing rates for 2%/2 mm and 3%/3 mm criteria, respectively. In this work, we evaluated the feasibility of electronic 2D detector arrays for CyberKnife measurements. The mentioned characteristics for the two detector types and geometries translate into the need for dosimetry system‐specific gamma evaluation criteria when employing the two systems for CyberKnife delivery measurements. For ArcCHECK, the 3%/3 mm gamma passing rate metric is insensitive for detecting types of delivery errors (5, 6) and this phenomenon is observed in all the recruited cases (Table 2). Therefore, 2%/2 mm appears to be more feasible for CyberKnife patient‐specific QA with ArcCHECK. As addressed in the literature^38^, ^39^, ^40^, ^41^ for MatriXX, the 3%/3 mm criterion is insensitive for detecting many forms of VMAT/IMRT delivery error. However, it is shown in this work that, considering the detector response to the small beams and the large incident beam angle created by the CyberKnife delivery, the 3%/3 mm criterion is more adequate for CyberKnife patient‐specific QA with MatriXX.

In addition to measure and compare the dose in the peripheral region, the 3DVH (Sun Nuclear Corp.) software can also reconstruct the 3D dose based on the measured peripheral dose distribution. However, this function is not suitable for CyberKnife patient‐specific QA because many beams could be completely (5 mm and 7.5 mm) or partially (all other cone sizes) missed due to the big spacing between the diode detectors. This will bring uncertainties to the interpolation and prediction of the beam intensity for 3D dose construction. Some measurement techniques, such as the high‐density merge option in the ArcCHECK SNC Patient software, could mitigate the disadvantage of large distance between the diode detectors by acquiring two sets of the data with ArcCHECK phantom shift 5 mm and rotated 2.7° between the two acquisitions. These measurement techniques could improve the accuracy of the reconstructed 3D dose but at the same time they also increase the workload.

There are other dosimetry methods that have the potential for CyberKnife delivery measurements. 2D film dosimetry offers superior spatial resolution for small field measurements. However, as mentioned in the Introduction, the resulting dose distributions are sensitive to the handling method and it gives a cumulated dose results, which fails to track when the delivery error happened. On the other hand, despite the limited spatial resolution, most electronic dosimetry systems are capable for time‐resolving analysis, which can pick up the delivery error of any specific beam. A recently developed scintillation phantom, which allows a 3D QA in a beam‐by‐beam basis, can also be an option for CyberKnife patient‐specific QA^(42)^ in the future.

## V. CONCLUSIONS

This study demonstrates that both planar and cylindrical detectors are feasible for CyberKnife extracraninial patient‐specific QA. With the smaller detector size, peripheral helical detector arrangement, along with the enhanced ability to detect small dose errors, rotational errors, and random errors, allows the cylindrical diode array to be preferable for both small and large target cases. However, the current form of planar ion chamber array is only feasible for large target cases due to its larger detector size. Thus, further research and development on the planar ion chamber systems are needed for small target measurements. It is preferable for both systems to increase the number of detectors per area or to develop measurement technique that can increase the resolution of the measured dose distribution. New phantom designs are also desired for CyberKnife intracranial treatment QA because the current designs of both dosimetry systems are not suitable for intracranial treatments with considerations of the safety clearance, radiation damage to the electronic part, and the increased workload with alternating the phantom setup orientations for measuring both transverse and vertex beams.

## ACKNOWLEDGMENTS

The authors would like to thank Sun Nuclear, Corp for the technical assistances; Dr. Yulong Yan (University of Texas Southwestern Medical Center, TX) for his assistance with the DICOM‐3 file format; Dr. Jiajin Fan (Fox Chase Cancer Center, PA) for constructive suggestions.

## References

[1] American College of Radiology (ACR) and American Society for Therapeutic Radiology. ACR–ASTRO practice parameter for the performance of stereotactic radiosurgery. Amended 2014 (Resolution 39). Available from: http://www.acr.org/~/media/ACR/Documents/PGTS/guidelines/Stereotactic_Radiosurgery.pdf

[2] Dieterich S , Cavedon C , Chuang CF , et al. Report of AAPM TG 135: quality assurance for robotic radiosurgery. Med Phys. 2011;38(6):2914–36. 21815366 10.1118/1.3579139

[3] Klein EE , Hanley J , Bayouth J , et al. Task Group 142 report: quality assurance of medical accelerators. Med Phys. 2009;36(9):4197–212. 19810494 10.1118/1.3190392

[4] Zeidan OA , Stephenson SA , Meeks SL , et al. Characterization and use of EBT radiochromic film for IMRT dose verification. Med Phys. 2006;33(11):4064–72. 17153386 10.1118/1.2360012

[5] Kairn T , Hardcastle N , Kenny J , Meldrum R , Tome WA , Aland T . EBT2 radiochromic film for quality assurance of complex IMRT treatments of the prostate: micro‐collimated IMRT, RapidArc, and TomoTherapy. Australas Phys Eng Sci Med. 2011;34(3):333–43. 21748444 10.1007/s13246-011-0087-z

[6] Wilcox EE and Daskalov GM . Evaluation of GAFCHROMIC EBT film for Cyberknife dosimetry. Med Phys. 2007;34(6):1967–74. 17654899 10.1118/1.2734384

[7] Gallo JL , Kaufamn I , Powell R , et al. Single‐fraction spine SBRT end‐to‐end testing on TomoTherapy, Vero, TrueBeam, and CyberKnife treatment platforms using a novel anthropomorphic phantom. J Appl Clin Med Phys. 2015;16(1):5120. 25679169 10.1120/jacmp.v16i1.5120 PMC5689980

[8] Jursinic PA and Nelms BE . A 2‐D diode array and analysis software for verification of intensity modulated radiation therapy delivery. Med Phys. 2003;30(5):870–79. 12772995 10.1118/1.1567831

[9] Poppe B , Blechschmidt A , Djouguela A , et al. Two‐dimensional ionization chamber arrays for IMRT plan verification. Med Phys. 2006;33(4):1005–15. 16696477 10.1118/1.2179167

[10] Herzen J , Todorovic M , Cremers F , et al. Dosimetric evaluation of a 2D pixel ionization chamber for implementation in clinical routine. Phys Med Biol. 2007;52(4):1197–208. 17264380 10.1088/0031-9155/52/4/023

[11] Keeling VP , Ahmad S , Jin H . A comprehensive comparison study of three different planar IMRT QA techniques using MapCHECK 2. J Appl Clin Med Phys. 2013;14(6):4398. 24257283 10.1120/jacmp.v14i6.4398 PMC5714623

[12] Gloi AM , Buchana RE , Zuge CL , Goettler AM . RapidArc quality assurance through MapCHECK. J Appl Clin Med Phys. 2011;12(2):3251. 21587169 10.1120/jacmp.v12i2.3251 PMC5718678

[13] Jursinic PA , Sharma R , Reuter J . MapCHECK used for rotational IMRT measurements: step‐and‐shoot, TomoTherapy, RapidArc. Med Phys. 2010;37(6):2837–46. 20632595 10.1118/1.3431994

[14] Dobler B , Streck N , Klein E , Loeschel R , Haertl P , Koelbl O . Hybrid plan verification for intensity‐modulated radiation therapy (IMRT) using the 2D ionization chamber array I'mRT MatriXX—a feasibility study. Phys Med Biol. 2010;55(2):N39–N55. 20023326 10.1088/0031-9155/55/2/N02

[15] Hussein M , Adams EJ , Jordan TJ , Clark CH , Nisbet A . A critical evaluation of the PTW 2D‐ARRAY seven29 and OCTAVIUS II phantom for IMRT and VMAT verification. J Appl Clin Med Phys. 2013;14(6):4460. 24257288 10.1120/jacmp.v14i6.4460 PMC5714639

[16] Letourneau D , Publicover J , Kozelka J , Moseley DJ , Jaffray DA . Novel dosimetric phantom for quality assurance of volumetric modulated arc therapy. Med Phys. 2009;36(5):1813–21. 19544800 10.1118/1.3117563

[17] Yan G , Lu B , Kozelka J , Liu C , Li JG . Calibration of a novel four‐dimensional diode array. Med Phys. 2010;37(1):108–15. 20175471 10.1118/1.3266769

[18] Kozelka J , Robinson J , Nelms B , Zhang G , Savitskij D , Feygelman V . Optimizing the accuracy of a helical diode array dosimeter: a comprehensive calibration methodology coupled with a novel virtual inclinometer. Med Phys. 2011;38(9):5021–32. 21978046 10.1118/1.3622823

[19] Petoukhova AL , van Egmond J , Eenink MG , Wiggenraad RG , van Santvoort JP . The ArcCHECK diode array for dosimetric verification of HybridArc. Phys Med Biol. 2011;56(16):5411–28. 21804180 10.1088/0031-9155/56/16/021

[20] Fakir H , Gaede S , Mulligan M , Chen JZ . Development of a novel ArcCHECK insert for routine quality assurance of VMAT delivery including dose calculation with inhomogeneities. Med Phys. 2012 Jul;39(7):4203–08. 10.1118/1.4728222 22830753

[21] Li G , Zhang Y , Jiang X , et al. Evaluation of the ArcCHECK QA system for IMRT and VMAT verification. Phys Med. 2013;29(3):295–303. 22583979 10.1016/j.ejmp.2012.04.005

[22] Lin MH , Koren S , Veltchev I , et al. Measurement comparison and Monte Carlo analysis for volumetric‐modulated arc therapy (VMAT) delivery verification using the ArcCHECK dosimetry system. J Appl Clin Med Phys. 2013;14(2):3929. 23470927 10.1120/jacmp.v14i2.3929 PMC5714369

[23] Bedford JL , Lee YK , Wai P , South CP , Warrington AP . Evaluation of the Delta4 phantom for IMRT and VMAT verification. Phys Med Biol. 2009;54(9):N167–N176. 19384007 10.1088/0031-9155/54/9/N04

[24] Ezzell GA , Burmeister JW , Dogan N , et al. IMRT commissioning: multiple institution planning and dosimetry comparisons, a report from AAPM Task Group 119. Med Phys. 2009;36(11):5359–73. 19994544 10.1118/1.3238104

[25] Low DA , Harms WB , Mutic S , Purdy JA . A technique for the quantitative evaluation of dose distributions. Med Phys. 1998;25(5):656–61. 9608475 10.1118/1.598248

[26] IBA Dosimetry . I'mRT MatriXX user's guide. Schwarzenbruck, Germany: IBA Dosimetry BmbH.

[27] Sun Nuclear. ArcCHECK user's guide. Melbourne, FL: Sun Nuclear; 2009.

[28] Accuray. Physics essentials guide. Sunnyvale, CA: Accuray; 2006.

[29] Ma CA , Deng J , Fan J . Implementation of Monte Carlo Dose calculation for CyberKnife treatment planning. Monte Carlo Techniques in Radiotherapy Delivery and Verification: Third McGill International Workshop; Montreal, Canada. J Phys Conf Series. 2008;102(1):012016.

[30] Sharma SC , Ott JT , Williams JB , Dickow D . Clinical implications of adopting Monte Carlo treatment planning for CyberKnife. J Appl Clin Med Phys. 2010;11(1):3142. 20160699 10.1120/jacmp.v11i1.3142 PMC5719782

[31] Alfonso R , Andreo P , Capote R , et al. A new formalism for reference dosimetry of small and nonstandard fields. Med Phys. 2008;35(11):5179–86. 19070252 10.1118/1.3005481

[32] Sharma SC , Ott JT , Williams JB , Dickow D . Commissioning and acceptance testing of a CyberKnife linear accelerator. J Appl Clin Med Phys. 2007;8(3):2473. 17712305 10.1120/jacmp.v8i3.2473 PMC5722603

[33] Veltchev I , Lin M , Price, R , Ma CM . Patient‐specific IMRT QA for large volume CyberKnife plans using MatriXX [abstract]. Med Phys. 2013;40(6):243.

[34] Li QL , Deng XW , Chen LX , Huang XY , Huang SM . The angular dependence of a 2‐dimensional diode array and the feasibility of its application in verifying the composite dose distribution of intensity‐modulated radiation therapy. Chin J Cancer. 2010;29(6):617–20. 20507735 10.5732/cjc.009.10592

[35] Wolfsberger LD , Wagar M , Nitsch P , Bhagwat MS , Zygmanski P . Angular dose dependence of Matrixx™ and its calibration. J Appl Clin Med Phys. 2010;11(1):3057. 20160692 10.1120/jacmp.v11i1.3057 PMC5719776

[36] Feygelman V , Zhang G , Stevens C , Nelms BE . Evaluation of a new VMAT QA device, or the “X” and “O” array geometries. J Appl Clin Med Phys. 2011;12(2):3346. 21587178 10.1120/jacmp.v12i2.3346 PMC5718675

[37] Sudahar H , Kurup PG , Murali V , Velmurugan J . Dose linearity and monitor unit stability of a G4 type cyberknife robotic stereotactic radiosurgery system. J Med Phys. 2012;37(1):4–7. 22363106 10.4103/0971-6203.92714 PMC3283915

[38] Kruse JJ . On the insensitivity of single field planar dosimetry to IMRT inaccuracies. Med Phys. 2010;37(6):2516–24. 10.1118/1.3425781 20632563

[39] Nelms BE , Zhen H , Tome WA . Per‐beam, planar IMRT QA passing rates do not predict clinically relevant patient dose errors. Med Phys. 2011;38(2):1037–44. 21452741 10.1118/1.3544657 PMC3188652

[40] Nelms BE , Chan MF , Jarry G , et al. Evaluating IMRT and VMAT dose accuracy: practical examples of failure to detect systematic errors when applying a commonly used metric and action levels. Med Phys. 2013;40(11):111722. 24320430 10.1118/1.4826166 PMC8353583

[41] Stasi M , Bresciani S , Miranti A , Maggio A , Sapino V , Gabriele P . Pretreatment patient‐specific IMRT quality assurance: a correlation study between gamma index and patient clinical dose volume histogram. Med Phys. 2012;39(12):7626–34. 23231310 10.1118/1.4767763

[42] Scintillating phantom offers CyberKnife QA [Internet article]. Med Phys Web. IOP Publishing; 2014 Available from: http://medicalphysicsweb.org/cws/article/research/57638

